# Molecular perspectives on life-history evolution in Bruchinae seed beetles

**DOI:** 10.1590/1678-4685-GMB-2025-0211

**Published:** 2026-07-10

**Authors:** Bruno de Oliveira Cruz, Alessandro de Mello Varani, Sílvia de Oliveira Miranda, Juliana Ramos Martins, Daniel Guariz Pinheiro, Angel Roberto Barchuk

**Affiliations:** 1 Universidade Federal de Alfenas (UNIFAL-MG), Instituto de Ciências Biomédicas, Departamento de Biologia Celular e do Desenvolvimento, Alfenas, MG, Brazil.; 2 Universidade Estadual Paulista (UNESP), Faculdade de Ciências Agrárias e Veterinárias, Departamento de Biotecnologia Agropecuária e Ambiental, Jaboticabal, SP, Brazil.

**Keywords:** Bruchinae, seed beetle, life-history trait, pest, genome

## Abstract

Phytophagous beetles represent one of the largest groups of plant-feeding insects. With about 135,000 described species, they exploit seeds, leaves, stems, and wood. Within this diversity, the subfamily Bruchinae has a distinctive ecological niche: larvae develop inside legume seeds. These beetles are major agricultural pests, responsible for up to 20% losses in legume grain value. This yield loss represents a severe problem, as legumes provide essential dietary protein for roughly 75% of people in developing countries. Despite their importance, the molecular foundations of life-history traits facilitating Bruchinae adaptation to seeds have only recently begun to be explored, leaving current knowledge sparse and fragmented. This review synthesizes current knowledge of the molecular mechanisms underlying the nutritional and digestive challenges faced by beetles during seed infestation, reproduction, and development, as well as during host shifts to novel or unusual legumes. Drawing on well-established examples, it examines key processes involved in the co-evolutionary dynamics between phytophagous insects and their host plants, while highlighting recent genomic advances that are accelerating discovery in these fields. Collectively, the review integrates both historical and contemporary molecular perspectives on life-history evolution in Bruchinae seed beetles.

## Introduction

Phytophagous beetles, predominantly belonging to the superfamilies Chrysomeloidea and Curculionoidea, together with lepidopterans and hemipterans, constitute the largest assemblage of plant-feeding insects (McKenna et al., 2019). These lineages form a phylogenetically diverse group that has evolved distinct life-history traits enabling the exploitation of seeds, leaves, stems, and wood, resulting in substantial agricultural losses. The subfamily Bruchinae, associated with legumes (Fabaceae), exhibits a unique relationship with seeds, where larvae develop after oviposition. Most Bruchinae females deposit their eggs on the surface of a host seed or pods. After the embryo develops, the larva hatches, penetrates the seed coat or the pod wall and the seed coat, and feeds on the cotyledons and embryonic regions of the seed until pupation. After that, the adults chew an exit hole and emerge, typically after 26 days of development at 30-35 °C. They undergo a brief 10-day reproductive period and then die (e.g. Corrêa et al., 2020, 2021).

The feeding regime of members of the subfamily Bruchinae depends on the developmental stage; larvae feed exclusively on seeds, whereas adults may feed on pollen and nectar (mainly “field Bruchinae”, income breeders, that use resources acquired during the breeding season to produce the offspring, rather than using previously stored reserves; Southgate, 1979). Larvae have been reported to feed on the seeds of at least 36 plant families, though more than 80% feed on legumes (Teixeira et al., 2008; Ribeiro-Costa and Almeida, 2012). A notable example of a non-leguminous feeder is the Brazilian beetle *Pachymerus nucleorum*. This species oviposits on developing infrutescences of *Allagoptera arenaria* (Arecaceae) palm tree thus causing enormous economic loss (Lacerda et al., 2024). Many species thus directly impact the economy by feeding on grain legumes, reducing the protein available to humans. Additionally, the beetles excrete nitrogenous waste as uric acid in the form of dry crystalline powder inside the seeds, making them hazardous for human consumption (Southgate, 1979; Das et al., 2021b). Others destroy seeds of leguminous trees and shrubs that, while not economically valuable, help prevent desertification. Mismanagement, like overgrazing, allows these seed-destroying organisms to hinder plant regeneration, ultimately harming agriculture, as seen in parts of Africa and the Middle East (Lamprey et al., 1974; Kergoat et al., 2008). Species of Bruchinae breed on every continent except Antarctica, with the largest number of species living in the tropical regions of Asia, Africa, and Central and South America. In Brazil, for instance, *Zabrotes subfasciatus, Acanthoscelides obtectus,* and *Callosobruchus maculatus* are the main bean pests (Ribeiro-Costa and Almeida, 2012). 

The successful exploitation of diverse Fabaceae hosts by Bruchinae beetles reflects a remarkable capacity to overcome nutritional, chemical, and developmental challenges through adjustments of their digestive and reproductive molecular machinery. However, despite their substantial ecological and economic significance, the molecular mechanisms governing the development of key life-history traits that enable Bruchinae adaptation to diverse leguminous seeds remain largely unexplored, leaving current knowledge sparse and fragmented. This review synthesizes current knowledge on the molecular landscape underlying nutrition, reproduction, and development in Bruchinae, with particular emphasis on mechanisms associated with novel or unusual host use and recent advances in Bruchinae genomics. Elucidating these molecular mechanisms would reveal novel targets for sustainable pest control strategies and provide valuable insights into the co-evolutionary dynamics between phytophagous insects and their host plants.

## Nutrition and digestive challenges - the arms race between Bruchinae and seeds

Digestion is the process by which food is broken down into smaller molecules that can be absorbed by gut cells. In insects, this process is tightly regulated by digestive enzymes, whose activity depends on their specific localization within the gut. In Bruchinae beetles -primarily capital breeders that do not feed during adulthood- all nutritional demands must be met during the larval stage. These larvae develop entirely within legume seeds, which, while rich in proteins and starch, also contain a variety of defensive compounds such as enzyme inhibitors, lectins, and other anti-nutritional factors. To survive and grow in this chemically hostile environment, larvae must adapt at the molecular level (Moon et al., 2004; Terra and Ferreira, 2012; Das et al., 2021 a). Such adaptations are critical, as larval diet quality directly influences key life-history traits. Factors such as adult body size, fecundity, and reproductive timing depend on these nutrients and are central to evolutionary fitness.

## The challenges of protein and carbohydrate digestion

**Figure 1 -:**
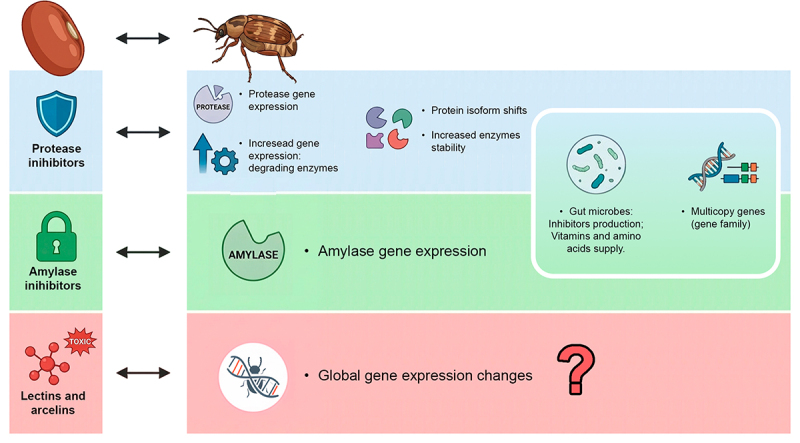
Evolutionary adaptations of Bruchinae for host plant seed exploitation and resistance to plant defence responses. Left panel: Plant responses to larval attempts to exploit seeds as a nutritional resource and developmental site. Right panel: Adaptive strategies evolved by Bruchinae beetles to utilize plant seeds and to overcome or circumvent plant defence mechanisms. ?=indicates mechanisms that remain poorly understood and require further investigation.

**Figure 2 -:**
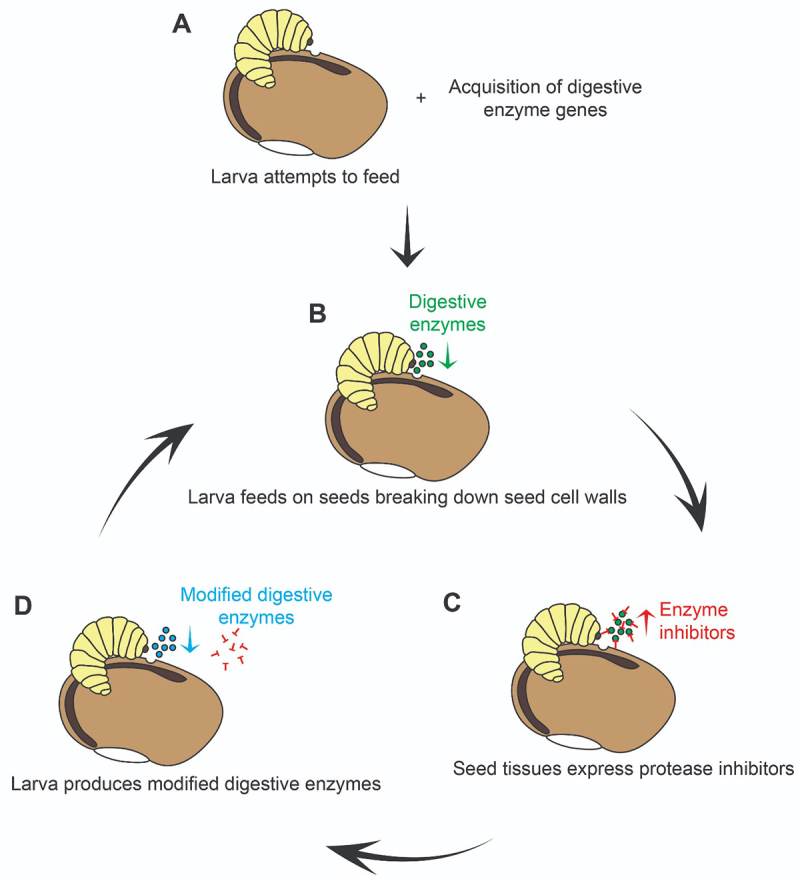
A schematic diagram showing the co-evolutionary arms race between Bruchinae species and Fabaceae seeds. A) Larvae attempt to feed and develop within seeds but face difficulties due to the absence of enzymes capable of digesting seed cell wall components. B) Larvae successfully feed on seeds after acquiring genes from associated microorganisms that encode enzymes capable of breaking down seed cell walls. Larvae can also take advantage of the digestive service provided by their gut microbiome. C) Seed tissues respond by expressing enzyme inhibitors, which interfere with the larvae’s digestive enzymes and hinder nutrient acquisition. D) Larvae counteract these inhibitors by producing biochemically modified digestive enzymes that remain functional despite the plant’s defensive compounds (see, for instance, Ehrlich and Raven, 1964; Zu et al., 2020). In addition to digestive enzymes and their respective inhibitors, this type of arms race may also involve a variety of “weapons”, such as toxic metabolites produced by plants and even microorganisms.

One of the primary molecular challenges faced by Bruchinae larvae is the presence of protease inhibitors (PIs) in seeds. These compounds target diverse digestive enzymes in the beetle midgut, such as serine (Magalhães et al., 2007), cysteine, and aspartic proteases (Zhu‐Salzman et al., 2003). These proteinases are essential for digestion. In response, Bruchinae have evolved robust counter-defensive strategies, most of which have been characterized in *C. maculatus*. Available evidence indicates that these beetles possess multigene families encoding digestive proteases, suggesting a capacity to alternate among different enzyme types in response to variable PIs profiles (Zhu‐Salzman *et al*., 2003). Moreover, dietary exposure to cysteine PIs such as soyacystatin N (scN) elicits a strong adaptive response beginning in the fourth larval instar. This response includes increased midgut proteolytic activity mediated by the upregulation of specific cysteine proteases, qualitative shifts in expressed isoforms (Zhu‐Salzman *et al*., 2003; Nogueira et al., 2012), and enhanced enzyme stability (Ahn et al., 2007). For example, transcripts encoding CmCP-B isoforms increase more than 100-fold relative to CmCP-A in scN-adapted insects (Ahn *et al*., 2004). Collectively, the evidence indicates that Bruchinae have evolved four complementary quantitative and qualitative mechanisms to overcome plant defensive barriers imposed by protease inhibitors (PIs), illustrating the staggered nature of the evolutionary arms race between larvae and seeds: (i) diversification of multigene protease families targeting plant; (ii) transcriptional upregulation of genes encoding digestive enzymes; (iii) modulation of protease isoform expression; and (iv) increased biochemical stability of enzyme molecules (Figures 1 and 2).

In addition to protein digestion, Bruchinae larvae must process carbohydrate-rich seed tissues. They achieve this through the production of α-amylases, α-glucosidases, β-glucosidases, and mannanases. In species such as *A. obtectus* and *Z. subfasciatus*, midgut α-amylases are naturally resistant to the α-amylase inhibitor αAI-1 found in common beans (*Phaseolus vulgaris*), allowing normal larval development in its presence. Like their defence against PIs, some species also produce serine proteases capable of degrading αAI-1, thereby ensuring uninterrupted carbohydrate digestion (Ishimoto and Chrispeels, 1996; Silva et al., 2001) (Figure 1). Interestingly, transgenic legumes expressing αAI-1 (and its homolog αAI-2) are effective against Old World Bruchinae species like *C. maculatus, C. chinensis,* and *Bruchus pisorum*, whose amylases are sensitive to the inhibitor. However, New World species (e.g. *A. obtectus* and *Z. subfasciatus*) remain unaffected due to preadapted, inhibitor-resistant enzyme phenotypes (Chrispeels *et al*., 1998; Busch et al., 2017; Brascher et al., 2024).

## The challenges of lectins and arcelins

Beyond targeted inhibitors, legume seeds deploy broader defence proteins such as lectins -carbohydrate-binding molecules that attach to glycoproteins on insect midgut cells. These interactions can disrupt the peritrophic matrix and brush border, induce oxidative stress, trigger apoptosis, and interfere with nutrient uptake (Jain et al., 2022). Some lectins may also cross the gut barrier into the haemolymph, further impairing larval metabolism (Napoleão et al., 2019). The lectin PF2 from *Olneya tesota* seeds, for example, has insecticidal activity against *Z. subfasciatus* and binds midgut proteins like α-amylase (acting as an αAI), actin, polyubiquitin, and arginine kinase -key players in energy metabolism and cellular integrity (Santimone et al., 2004; Lagarda-Diaz et al., 2016). Although molecular data on Bruchinae-specific responses to lectins remain limited (e.g. global shifts in gene expression patterns, Wang et al., 2015), general defence pathways such as detoxification enzyme induction and microbiome modulation likely contribute to resistance (Figure 1).

Closely related to lectins in structure, arcelins are another class of defence proteins present in wild varieties of *P. vulgaris* and *P. lunatus*. Although they lack carbohydrate-binding domains, arcelins share sequence homology and three-dimensional similarities with lectins (Karuppiah et al., 2018). Eight arcelin variants (27- 42 kDa) have been identified, many of which resist proteolysis and interact with midgut glycoproteins and membranes, leading to larval starvation. Some arcelin proteins have shown toxicity against *Z. subfasciatus* and *C. maculatus*, making them potential candidates for crop improvement strategies (Osborn et al., 1988; Karuppiah *et al*., 2018; Jain et al., 2022). Future work should further elucidate how lectins, arcelins, and related toxins perturb gut homeostasis in these insects, and how beetles counteract their toxicity (Figure 1).

## The helpful microbes

When larvae cannot rely solely on endogenous enzymes, they may benefit from microbial symbionts. In *C. maculatus*, a conserved strain of *Staphylococcus gallinarum* is transmitted between larval and adult stages, supplying B vitamins, amino acids (e.g., tyrosine for cuticle synthesis), and various digestive enzymes (Berasategui et al., 2021). Some Bruchinae may host microbes capable of degrading inhibitors or supplementing essential nutrients, though direct molecular characterization is lacking (Figure 1). Additionally, evidence suggests that several enzymes currently expressed in Bruchinae originated from microbial genes, indicating an evolutionary integration of host and symbiont metabolic capabilities (Kirsch et al., 2014). Following their acquisition, these genes underwent functional diversification within the beetle lineage, representing a pivotal innovation that facilitated the evolution of herbivory. Early Bruchinae likely exploited these enzymes to degrade seed tissues efficiently. In response, host plants evolved enzyme inhibitors that constrained beetle digestion, thereby imposing selective pressure on beetles to modify enzyme structure through increased stability, shifts in isoform expression, or changes in amino-acid sequence. This reciprocal pattern of adaptation constitutes a classic evolutionary arms race between Fabaceae plants and Bruchinae beetles (Figure 2). The interplay between these symbiotic functions and nutrient-sensing pathways -such as Insulin/IGF-like Signalling (IIS) and Target-of-Rapamycin (TOR)- remains understudied. These conserved pathways likely mediate responses to nutrient availability, modulating enzyme secretion, growth, and development. However, their role in Bruchinae and the phylogenetic distribution of microorganisms-derived digestive enzyme-coding genes requires further investigation.

As shown above, legume seeds deploy a sophisticated arsenal of evolutionarily refined defensive proteins to deter Bruchinae pests, for all but one of which beetles have evolved molecular countermeasures -lectins seem to be a notable exception (Figure 1). Yet, despite progress in understanding these interactions, developing truly pest-resistant crops remains elusive. A central challenge is the Bruchinae beetles’ remarkable adaptability, which enables them to overcome a broad range of plant defences. Protease inhibitors, long considered promising biocontrol agents, are now being re-evaluated in the context of modern technologies. Multigene and plastid engineering, functional proteomics, and combination approaches -such as recombinant inhibitors with RNAi or CRISPR/Cas9-based gene disruption- offer new avenues for durable resistance (Singh et al., 2020). Nonetheless, the coevolutionary arms race between plants and herbivores is likely to persist, underscoring the need for integrative strategies that anticipate and mitigate insect adaptation. Together, these mechanisms illustrate how reciprocal selective pressures shape both Bruchinae physiology and legume defence systems, thereby establishing the molecular foundation for the additional adaptive traits discussed in the following sections.

## Reproduction and Development

In insects, the fat body is a central metabolic organ that plays a key role in energy storage and the regulation of reproduction in adults (Klowden and Palli, 2023). In Bruchinae, larvae accumulate large energy reserves in this tissue; however, little is known about how and when this capacity is established. In particular, the earliest developmental stages -especially embryogenesis- remain poorly understood, as most existing studies focus on later stages such as fully formed larvae and pupae (Howe and Currie, 1964; Southgate, 1979; Rodríguez-Quiroz et al., 2000; Eady et al., 2007), or on reproductive behaviour and oviposition strategies (Pimbert, 1987; Pimbert and Pouzat, 1988; Fox, 1993; Savalli and Fox, 1999; Teixeira and Zucoloto, 2003, 2012; Teixeira *et al*., 2008, 2009; Corrêa et al., 2020, 2021). This lack of data hampers an integrated understanding of development and internal regulatory mechanisms. To address these limitations, molecular tools are crucial. They allow us to reveal how external signals are converted into specific endocrine and genetic responses.

## Oviposition plasticity and nutritional strategies

In *Z. subfasciatus*, females adjust both the number and the size of their eggs according to resource availability. When seeds are abundant, they produce smaller eggs in greater numbers to increase offspring quantity. Under scarcity, however, females prioritize larger eggs. Such a shift represents a higher individual investment and potentially greater survival probability for each descendant (Teixeira et al., 2009). Plasticity also modulates oviposition site selection. Females usually avoid depositing eggs on occupied seeds. However, under high density, they relax this selectivity. This behavioural plasticity leads to indiscriminate use of substrates, a behaviour known as “egg dumping”, typical of intense competition (Teixeira *et al*., 2016).

Responses to resource availability are deeply linked to how nutrients sustain reproduction. Species such as *Z. subfasciatus* are classically described as capital breeders. They rely on reserves accumulated in the fat body during larval development (Corrêa et al., 2020; Miranda et al., 2025). Vitellogenesis begins as early as the pupal stage, ensuring that many females are able to oviposit just a few hours after emergence (Miranda *et al*., 2025). This reproductive strategy favours rapid colonization of new environments. In contrast, species such as *B. pisorum* follow the income breeding model. These adults must feed on pollen before starting oviposition (Aznar-Fernández et al., 2020). This strategy allows for adjustments to immediate conditions but slows down the process. The coexistence of these models suggests that differences in hormonal regulation underlie this strategic diversity.

## Chemical signalling and hormonal control

**Figure 3 -:**
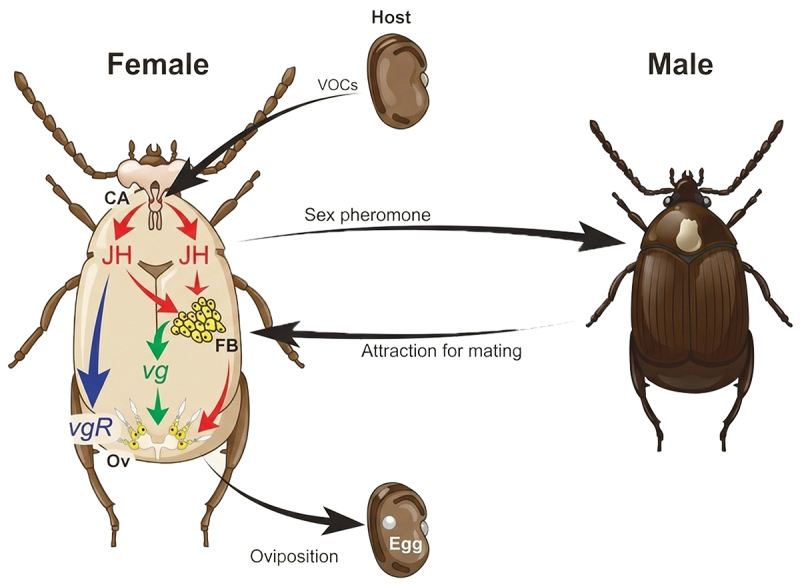
Proposed working model of oviposition regulation in capital breeders Bruchinae The proposed increase in juvenile hormone (JH) synthesis following female exposure to host seeds, as well as the chemical nature of the sex pheromone involved (homofarnesals and monoamines), require further experimental validation. VOCs= volatile organic compounds; CA= *corpora allata*; FB= fat body; Ov= ovaries; JH= juvenile hormone; Vg= vitellogenin; VgR= vitellogenin receptor. Modified from Miranda et al. (2025), with information from Shimomura et al. (2008) and Yamane (2014).

Host seeds directly modulate the reproductive physiology of Bruchinae. Contact with mature *P. vulgaris* seeds acts as a strong stimulus for oogenesis and oviposition. Conversely, immature pods induce copulation only, without triggering egg laying. These effects are mediated, at least in part, by seed-emitted volatile organic compounds (VOCs), which function as chemical signals that attract insects and likely stimulate increased sex pheromone production in females, thereby reinforcing intraspecific communication (Pimbert and Pierre, 1983; Pimbert and Pouzat, 1988). Such behaviours reflect hormonal pathways connecting environmental signals to ovarian activation (Figure 3). However, the chemical nature of the sex pheromone involved (e.g., homofarnesals and monoamines) has so far been elucidated only for *Callosobruchus* (Shimomura et al., 2008), and further research is needed to determine how broadly these compounds are used across species.

In *Z. subfasciatus*, VOCs may act as early inducers of juvenile hormone (JH) synthesis (Pimbert, 1987; Pimbert and Pouzat, 1988; Capizzani et al., 2024). Drawing an analogy to model insects like *Drosophila melanogaster*, *Aedes aegypti*, and *Tribolium castaneum*, increased JH levels trigger a molecular cascade. This includes vitellogenin (vg) synthesis in the fat body and activation of the vitellogenin receptor (vgR) in the ovaries (Leyria, 2024). Notably, in *Z. subfasciatus*, this maturation process is rapid. Vitellogenesis begins in the pharate adult stage, allowing oviposition on the first day after emergence (Miranda et al., 2025). Ovarian morphology further facilitates this process. In Polyphaga, including Bruchinae, ovaries are telotrophic-meroistic (Büning, 1994; Leyria, 2024; Miranda *et al*., 2025). This ovarian architecture ensures a continuous molecular supply of RNAs and proteins to developing oocytes. In capital breeders, it facilitates the efficient mobilization of larval fat reserves for rapid oocyte growth.

## Social interactions and reproductive trade-offs

Beyond host seed cues, the social context acts as an additional regulator of reproductive processes. In *Z. subfasciatus*, females maintained with males exhibit faster ovarian activation. Isolated females show restricted cellular activity and slower vitellogenesis (Miranda et al., 2025). Interestingly, these effects are distinct at the molecular level: males primarily increase *vg* gene expression in the fat body, whereas seeds enhance *vgR* gene expression in the ovaries. Thus, social and environmental factors act complementarily to sustain vitellogenesis. These stimuli likely converge on the *corpora allata*, increasing JH release (Figure 3).

In *C. chinensis*, reproduction is further modulated by neurophysiological signals that mediate mate attraction and receptivity. As mentioned above, female-produced homofarnesenes function as sex pheromones that attract males, while neurotransmitters such as dopamine regulate female sexual receptivity (Shimomura et al., 2008). Importantly, comparative studies reveal that the magnitude and nature of these neurochemical effects vary across geographic populations, reflecting underlying genetic divergence in receptor sensitivity and neuroendocrine circuitry (Yamane, 2014).

While *C. chinensis* illustrates the role of neural and chemical signalling in reproductive regulation, *C. maculatus* emphasizes the physiological costs associated with mating. In this species, males possess armed genitalia that cause internal perforations in females, leading to reduced longevity and increased susceptibility to infection. In addition, compounds in the seminal fluid induce hormonal changes that further decrease female survival (Eady et al., 2007). Paradoxically, males also transfer large ejaculates that function as nuptial gifts, supplying nutrients that can temporarily offset these costs by prolonging female survival (Fox, 1993; Savalli and Fox, 1999).

## Conserved molecular pathways

The fat body functions as the central interface between nutritional signals and the reproductive program. Studies in model Coleopterans, such as *T. castaneum*, reveal an integrated regulatory system. JH signalling acts together with ecdysteroids (especially 20-hydroxyecdysone) and insulin-like peptides (ILPs). These hormones interact with conserved metabolic pathways: insulin/insulin-like signalling (IIS), target of rapamycin (TOR), and AMP-activated protein kinase (AMPK) (Parthasarathy et al., 2010; Leyria, 2024). The TOR pathway promotes protein translation, while IIS controls energy metabolism. Conversely, AMPK acts as an energy sensor, repressing vitellogenesis under scarcity (Klowden and Palli, 2023). In *T. castaneum*, nuclear receptors act as direct effectors of these cascades. Receptors such as E75, HR3, EcR, USP, and FTZ-F1 are indispensable for vitellogenesis. Their silencing via RNAi drastically reduces vitellogenin expression (Xu et al., 2010). Other receptors regulate embryogenesis success. These findings highlight that reproductive control relies on a nuclear network of transcription factors converting signals into genomic responses. Although specific data on Bruchinae is limited, it is plausible that similar molecular mechanisms ensure the integration of energy availability and ovarian activation.

In general, reproduction in Bruchinae results from a complex network of interactions among environmental stimuli, nutritional reserves, hormones, and regulatory genes. Although conserved pathways are well described in model insects (Parthasarathy et al., 2010; Xu et al., 2010; Klowden and Palli, 2023; Leyria, 2024), knowledge regarding the specific molecular biology of Bruchinae remains limited. Advancing in this direction is essential to understand how ovarian activation, hormonal regulation, and gene expression occur in this group. Only from this molecular basis will it be possible to establish broader connections with ecological and behavioural data, paving the way for an integrated view of Bruchinae reproductive biology and for the development of strategies for the management of these insects.

## Evolutionary dynamics of host expansion in Bruchinae

While the molecular machinery for digestion and reproduction allows Bruchinae to exploit their usual hosts efficiently, shifts to novel legumes impose severe stress on these systems. For phytophagous insects, host plants influence the development of advantageous molecular traits essential for their survival (Ashra and Nair, 2022). Bruchinae beetles are typically adapted to specific seeds of the Fabaceae family, which serve as their habitat, food source, and sites for reproduction and oviposition (Southgate, 1979). As oligophagous insects, they can infest other grains, but the significant variation in morphology and chemical compounds among different plant seeds presents a major challenge (Forbes et al., 2017). This challenge demands adaptive variations in the beetles to ensure survival. An insect’s choice of host plant is influenced by several factors. They vary from long-range cues like volatiles (triggers for ovarian activation, see Section 3) and visual signals to short-range cues such as tactile and gustatory contact with the plant’s surface (Knolhoff and Heckel, 2014). This selection process is also critically influenced by the insect’s life stage, physiological and reproduction conditions, learning ability, and genetic variation (Moreau et al., 2017).

## Phenotypic plasticity and life-history traits

Phenotypic plasticity can have important effects on the initial survival of individuals in a new environment and on the course of long-term evolutionary change. The ability of an individual’s genome to plastically adapt to new environmental factors is crucial for survival, specially under adaptation to a novel host. In general, Bruchinae beetles such as *A. obtectus* and *Z. subfasciatus* show remarkable plasticity of traits under host-shift. After selection, populations can adapt to unusual hosts by developing new life-history strategies. These changes include altered larval development time, increased body size, and earlier reproduction. Compared to ancestral populations, these insects may also show shorter lifespans and decreased trait plasticity (Savković et al., 2016; Cruz et al., 2025). In addition, females respond more plastically in terms of their body size and abdomen shape (having more alterations than males under adaptation), which suggests that body symmetry (index that includes abdomen shape) and body adaptation may be driven by sexual selection (Rončević et al., 2024; Cruz *et al*., 2025). 

**Figure 4 -:**
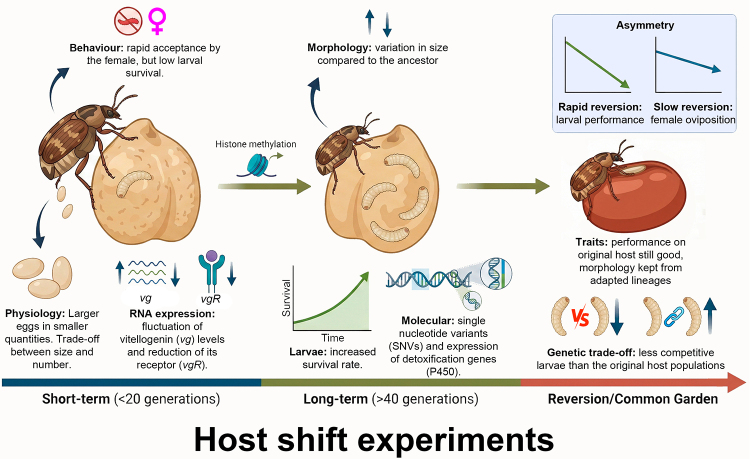
Evolutionary dynamics and molecular and physiological mechanisms associated with host expansion in Bruchinae. The schematic illustrates the adaptation stages of beetles (such as *Callosobruchus maculatus* and *Zabrotes subfasciatus*) when transferred from a usual host to an unusual one (e.g., chickpea). (Left - Short-term / <20 generations): Phase characterized by phenotypic plasticity. Although behavioural acceptance is rapid, larval survival is low. A physiological trade-off is observed with the production of fewer but larger eggs, fluctuation in vitellogenin (*vg*) expression, and reduction of its receptor (*vgR*), alongside epigenetic regulation via histone methylation. (Centre - Long-term / >40 generations): Increased larval survival rate and morphological variation (body size) occur. Molecular mechanisms include the selection of single nucleotide variants (SNVs) and expression of detoxification genes (e.g., *P450*). (Right - Reversion/Common Garden): Returning to the usual host reveals adaptive asymmetry. Larval performance reverses rapidly, while the female’s oviposition preference reverses slowly and morphology traits acquired during adaptation are retained. A genetic trade-off is evident, where adapted lineages are less competitive in the usual host compared to ancestral populations.

Bruchinae females exhibit remarkable plasticity in egg laying strategies, as discussed in Section 3. Under host-shift scenarios, this plasticity becomes a critical survival determinant. For instance, populations adapting to unusual hosts often prioritize egg size over number to maximize larval energy reserves involved in detoxifying novel defences (Figure 4). This pattern can be observed across many species: *S. limbatus* (Czesak and Fox, 2003), *C. maculatus* (Messina and Fry, 2003; Fox and Messina, 2018) and *Z. subfasciatus* (Teixeira et al., 2009; Cruz et al., 2025). Egg-size plasticity appears to be an ancestral trait that played a key role in the dietary expansion of Bruchinae, enabling the colonization of a wide range of host plants. By influencing larval performance, this plasticity subsequently shapes major life-cycle traits, including fecundity, larval development, adult size, and the sex ratio of the progeny (Amarillo-Suárez and Fox, 2006; Campan and Benrey, 2006).

## Genetic impact on host shifts

Given that reproductive success profoundly influences adaptation to novel hosts, the genetically determined traits are equally critical. This genetic constraint occurs because many adaptations have a genetic basis passed forward from parents to offspring. A critical challenge in adapting to novel hosts, for example, is the genetic independence between female acceptance and larval performance. Evidence in short-term experiments (<20 generations) with *C. maculatus* (Messina et al., 2009b) and *Z. subfasciatus* (Teixeira and Zucoloto, 2003; Cruz et al., 2025) indicates that oviposition preference can be rapidly induced or selected, but this does not guarantee offspring survival on unusual hosts (Figure 4). Frequently, females may accept hosts on which larval survival is extremely low (less than 2%), evidencing a mismatch between selection signals and actual biological fitness. Collectively, these results suggest a lack of genetic correlation between larval survival and host acceptance. However, host preference can be induced after a few generations (Messina *et al*., 2009b) or long-term selections (Figure 4) (Teixeira *et al*., 2008).

Host adaptation in Bruchinae can be induced and, under certain conditions, reversed, often involving evolutionary trade-offs. In *C. maculatus*, experimental populations that adapted to lentil -an unusual host- rapidly reverted to mung bean, the ancestral host. This reversal revealed genetic trade-offs associated with single-nucleotide variants (SNVs) that were positively selected during host adaptation but became deleterious when the selective environment changed (Figure 4) (Messina and Gompert, 2017). When populations are reverted to their usual host after a long-term selection of 62 generations, the frequency of these SNVs, which provides advantages on the lentil host, began to decrease. Despite showing this genetic trade-off, the beetle keeps its capacity of infesting the usual host and, when once again is transferred to lentils, the acceptance of the unusual host decay (Figure 4). Lineages in which the fecundity on lentils decays the most, show the smallest decay in survival of the offspring. Thus, genes that influence oviposition behaviour appear to be largely independent of genes that enhance larval performance (Messina and Gompert, 2017). Furthermore, reversibility is more evident and consistent in larval performance than in oviposition behaviour (Figure 4). Host adaptation may be asymmetric: acceptance of a novel host can fluctuate rapidly, while acceptance of the usual host often remains stable. 

Asymmetry in host acceptance by *C. maculatus* is well documented in genetic studies and provides a useful comparative model for research on other Bruchinae. During adaptation to novel hosts, this beetle can evolve a range of distinct traits. However, these traits might be useful only for a limited number of hosts and useless for others. This specificity is probably explained by the diversity of seed physical and chemical composition, which is an important determinant of survival rates for phytophagous insects. For example, it is remarkable that survival of *C. maculatus* on the unusual lentil host is an additive trait: hybrids survival is intermediate compared to the lentil and mung bean born parents, with the genetics of both influencing offspring fitness (Messina et al., 2009a). However, switching from lentil to other unusual hosts does not confer a survival advantage to this insect (Messina and Jones, 2009), suggesting that adaptation is host-induced and does not follow a standard pattern. To illustrate, the ability to develop on fava beans has a genetic basis, determined by a recessive autosomal gene, while the inability to develop is caused by a dominant gene. Adaptation to this unusual host results from low enzymatic activity that prevents the conversion of vicine into its toxic aglucone form (Desroches et al., 1997). Furthermore, lineages of *C. maculatus* transferred to cowpea can evolve to be significantly smaller than those maintained on the usual host after 40 generations (Figure 4). Larval competitiveness is also reduced: larvae exhibit avoidance behaviour rather than invading each other’s burrows and biting competitors (Figure 4). These differences are primarily genetic and not simply due to the immediate diet, as it can be demonstrated using reversion methods, returning *C. maculatus* to its usual host (a common garden approach, Figure 4) (Messina, 2004).

## Molecular tools explain adaptation

The repeatability of host-adaptation throughout research is a great way to understand how evolution works, especially in the genetic field. However, despite the high correlation between evolution and genetics (and its molecular basis), there is still a shortage of molecular biology studies focused on adaptations to novel hosts. New genomic tools, such as whole-genome sequencing and Quantitative Trait Locus (QTL) mapping, can be powerful allies for identifying the specific genes that drive evolutionary changes in host preference and performance. Through this method, the genetic basis of adaptation of *C. maculatus* to lentils was elucidated (Rêgo et al., 2020). It was shown that the heritability of the insect weight and development time is low to modest. This finding emerged from combined analyses of population genomics, genome-wide association mapping (GWA), and gene expression. These studies utilized DNA and RNA sequencing data from insect lines in long-term selection experiments. Gene expression differences are primarily due to evolved genetic differences rather than immediate plasticity to the host. Thus, parallel evolution is more evident in allele frequency changes than in the genetic architecture of performance traits (Figure 4). In addition, detoxification genes, such as those of cytochrome P450, likely play a significant role in the beetle adaptation to lentil (Figure 4) (Rêgo *et al*., 2020).

Molecular studies can keep unravelling possible molecular patterns for Bruchinae. Through these approaches, the adaptation factors in *Z. subfasciatus* host-shifting from its usual host, the common bean, to chickpea, unusual host, are being revealed*.* Researchers used RNA-Seq to analyse gene expression in young adult females after 10 generations of artificial selection. Populations selected for chickpea consumption showed a distinct transcriptional profile. In these groups, genes related to stimuli, signalling, and developmental processes were differentially expressed (Rodrigues et al., 2024). Specifically, chickpea populations show an upregulation of histone methylation genes (involved in epigenetic processes), suggesting a role for these genes in the insect’s adaptation to the novel host (Figure 4). *Z. subfasciatus* populations kept on bean also have higher expression levels of the genes polygalacturonase (*PGA*) and egalitarian (*egl*). Respectively, they encode a hydrolytic enzyme that degrades pectin in the plant cell wall and an RNA binding protein necessary for localization of several mRNAs. In addition, vitellogenin (*vg*) variants in both males and females from bean show increased transcription levels during selection on chickpea (Figure 4) (Rodrigues *et al*., 2024).

Molecular changes underpin reproductive adaptation in Bruchinae. For instance, host shifts in *Z. subfasciatus* are associated with altered expression of *vg* and *vgR* genes (Cruz et al., 2025). Females reared on chickpea exhibit increased oocyte retention alongside a reduced germarium size over 12 generations. During the early phases of adaptation, *vgR* transcript levels decline, whereas *vg* expression fluctuates (Figure 4). These patterns suggest that reproductive capacity may be impaired during host shifts, even when *vg* remains expressed. Thus, early adaptation to a novel host appears to be driven by reorganization of the gene expression landscape, which may underlie the reduced oviposition rates and overall fitness decline observed in adapting populations.

## Molecular changes under pest management protocols

Pest management protocols are now widely implemented in seed production and can exert significant molecular effects on Bruchinae. Thus, it is an important factor to be taken into consideration when discussing the beetles’ capacity of using a host, since they can adapt to deal with this problem. Analyses of the metabolic resistance of *C. maculatus* in West Africa showed that the insect’s survival to chemicals used in pest control is directly associated with enhanced detoxification capacity. There is an increase in enzymes such as esterases (naphthol acetate and para nitro phenyl acetate), glutathione S-transferase (GST), and oxidases when responding to insecticides such as pyrethroids and organophosphates. Thereby, enzymatic overproduction allows the insect to neutralize the toxic insecticide molecules before they can cause death (Zongo et al., 2020).

Exploring the biochemical mechanisms underlying Bruchinae control is essential for developing more sustainable pest management strategies at the molecular level. As an alternative to conventional chemical insecticides, botanical compounds can act as effective regulators of host use. Essential oils may act by competitively inhibiting vital nervous system enzymes like acetylcholinesterase (AChE) (Mattar et al., 2022). Molecular docking tools are fundamental for validating these processes. They allow precise simulations of how specific compounds, such as 1-epi-cadinol, bind to an enzyme’s active site. In addition to direct molecular efficacy, molecular tools revealed that the response of these beetles to chemical control is strongly mediated by their gut microbiota (see Section 2.4), predominantly Proteobacteria and Firmicutes. These bacterial phyla were identified through pyrosequencing 454 (V2-V3 regions of the 16S rRNA gene). The presence of these symbionts confers adaptive resistance to synthetic pesticides (e.g., dichlorvos) in Bruchinae, which can arise within as few as three generations. Essential oils act disruptively on the microbiota, drastically reducing bacterial populations and preventing the symbionts from assisting in the detoxification of the host (Akami et al., 2019).

In summary, Bruchinae beetles adapt to their hosts through a complex, multifaceted process driven by phenotypic plasticity, reproductive strategies, and underlying molecular factors. These insects rapidly adjust their life-history traits, such as development time, body size, and reproductive behaviour, to meet the challenges posed by chemically and physically distinct plant seeds. Trade-offs are a crucial adaptation that repeats across different species, demonstrating a predictable evolutionary response. Additionally, adaptation has a clear genetic basis, with the selection of heritable traits, like the ability to accept novel hosts and the activity of detoxification genes. Advances in molecular tools, particularly RNA sequencing, are beginning to elucidate the genetic mechanisms underlying these responses and are proving instrumental for understanding both pesticide resistance and interactions with symbiotic bacteria.

## Advances in genomics 

Access to genome sequences from a wide range of organisms has transformed the study of biological systems. In Bruchinae, however, molecular research until recently relied largely on targeted analyses of individual genes or gene products. These studies typically employed enzymatic assays combined with SDS-polyacrylamide gel electrophoresis or the isolation and sequencing of specific mRNAs through cDNA cloning (Gómez-Zurita and Galián, 2005). Despite these methodological limitations, such approaches yielded important insights, particularly into the expression and regulation of digestive enzymes in *Z. subfasciatus* (e.g. (Grossi de Sa and Chrispeels, 1997; Silva et al., 2001).

The first genome-scale resources available for Bruchinae consisted of mitochondrial genome (mitogenome) assemblies. The earliest complete mitogenomes were generated using PacBio sequencing and corresponded to four Bruchinae species, with reported genome sizes ranging from 24,496 bp in *C. chinensis* to 26,613 bp in *A. obtectus*. For example, the mitogenome of *C. maculatus* comprises 22 tRNA genes, 13 protein-coding genes, two ribosomal RNA genes, and a control region (Sayadi et al., 2017). Subsequent studies based on Illumina sequencing reported discrepancies in total mitogenome length and in the number of annotated tRNA genes (e.g. Zhang et al., 2018). Bruchinae mitogenomes are among the largest described in insects, a feature likely attributable to extensive non-coding regions -approximately 40% of the total genome- primarily due to two long intergenic spacers (Sayadi *et al*., 2017).

Fragments of mitochondrial DNA can occasionally escape the organelle and integrate into the nuclear genome, forming nuclear mitochondrial DNA segments (NUMTs) (Zhang and Hewitt, 1996). Because mitochondrial DNA is widely used in phylogenetic and biodiversity studies -owing to its maternal inheritance, region-specific evolutionary rates, and high copy number- the presence of NUMTs can confound evolutionary inference. NUMTs are widespread within the Chrysomelidae and exhibit both chromosomal and species specificity. They have been identified in several Bruchinae species, including *C. maculatus, Bruchidius siliquastri,* and *B. varius* (He *et al*., 2025).

**Figure 5 -:**
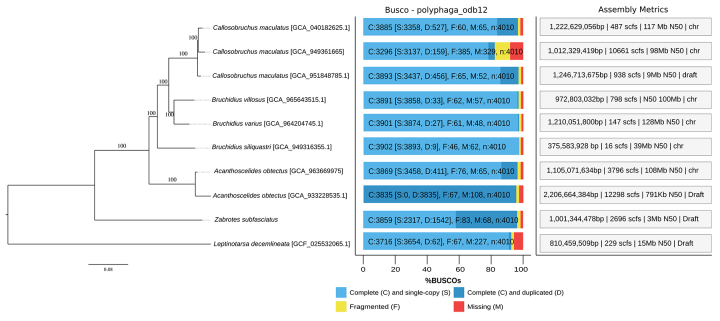
Phylogenetic tree inferred from 1,014 complete and single-copy BUSCO genes identified across all analysed genomes from Bruchinae species. The concatenated alignment (supermatrix) was generated using the supermatrix_only option of BUSCO_phylogenies (https://github.com/jamiemcg/BUSCO_phylogenomics) and the tree was reconstructed with IQ-TREE3 (iqtree3 -s SUPERMATRIX.phylip -p SUPERMATRIX.partitions.nex --alrt 1000 -B 1000 -T AUTO). *Leptinotarsa decemlineata* was used as the outgroup. Node support was assessed using 1,000 SH-aLRT replicates and 1,000 ultrafast bootstraps. Complementary panels display (i) the BUSCO completeness profile for each genome and (ii) the assembly metrics (genome size, N50, and assembly status [draft or chromossome-scale]) for the corresponding datasets. The *Zabrotes subfasciatus* genome was assembled with hifiasm v0.25.0-r726 using PacBio Sequel IIa HiFi reads. Data sources included a pooled dataset from a stock population of beetles (two SMRT cells), as well as additional samples from beetles reared for 10 generations on chickpea (*Cicer arietinum*, one SMRT cell) and beetles maintained on common bean (*Phaseolus vulgaris*, one SMRT cell). This Whole Genome Shotgun project has been deposited at DDBJ/ENA/GenBank under the accession JBRALV000000000. The version described in this paper is version JBRALV010000000.

More recently, nuclear genome assemblies have become available for several Bruchinae species (Figure 5). The first chromosome-level genome assembly within the subfamily was produced for *C. maculatus*, with a total size of approximately 1.01 Gb. This genome contains more than 21,000 annotated protein-coding genes (Sayadi et al., 2019; Arnqvist et al., 2024), is composed of approximately 70% repetitive sequences, and has a karyotype of n = 9 + X/Y (Arnqvist *et al*., 2015; Arnqvist *et al*., 2024; Lu et al., 2024). Draft or chromosome-scale assemblies are now also available for *B. siliquastri, B. villosus, B. varius,* and *A. obtectus*. In addition, a draft nuclear genome sequence has recently been generated for *Z. subfasciatus*, also totalling approximately 1.0 Gb, with a karyotype of n = 12 + X/Y (Takenouchi, 1972; Corrêa et al., 2008; Figure 5).

The genomes of *C. maculatus, A. obtectus,* and *Z. subfasciatus* show a high proportion of duplicated regions, particularly in one available *A. obtectus* assembly (GCA_933228535.1), which appears to be nearly entirely duplicated (Figure 5). These duplications may reflect genuine gene duplication events associated with evolutionary innovation. However, in the case of *A. obtectus* -whose chromosome number matches that of *C. maculatus* (Rozek et al., 1999) they are more likely to result from technical artifacts such as uncollapsed haplotigs or redundant assemblies (Guiglielmoni et al., 2021). When genuine, gene duplications can promote functional diversification, contributing novel or modified traits involved in the legume-beetle arms race and facilitating genomic reorganization through translocations, potentially accounting for variation in chromosome numbers among species.

Phylogenomic analyses based on nuclear genomic data place *Callosobruchus*/*Bruchidius* and *Acanthoscelides* on distinct branches of the Bruchinae phylogeny (Arnqvist et al., 2015; Chen et al., 2025), thereby supporting hypotheses regarding the geographic origins and distribution patterns of these genera (Southgate, 1979). This topology though contrasts with earlier phylogenies inferred from mitochondrial genomes, which suggested alternative relationships among these genera (Zhang et al., 2020). Within nuclear-based analyses, *Z. subfasciatus* consistently emerges as the most distantly related species among the Bruchinae taxa examined to date (Figure 5). A more comprehensive and systematically broad phylogenomic study will be essential to further clarify the relationships among genera and species within this group.

Early investigations of genome evolution in seed beetles revealed substantial interspecific variation in genome size, largely attributable to differences in non-coding heterochromatin. However, within species, genome size variation does not correlate with chromosome number, nor does it follow consistent patterns among closely related taxa. Notably, intraspecific genome size variation has been evolutionarily associated with key reproductive fitness traits, including female lifetime fecundity, male competitive fertilization success, and ventral genital spine length (Arnqvist et al., 2015). In *C. maculatus*, populations with larger genomes show enhanced capacity to buffer environmental stress, resulting in increased adult fitness under challenging conditions (Boman and Arnqvist, 2023).

Comparative genomic analyses further reveal rapid and extensive chromosomal rearrangements among Bruchinae species, potentially driven by lineage-specific amplification of long interspersed nuclear element (LINE) retrotransposons (Chen et al., 2025). These rearrangements appear to have facilitated translocation-based gene birth on the Y chromosome, whereby genes originally located on autosomes have been relocated and subsequently evolved male-biased expression (Chen *et al*., 2025). Bruchinae exhibit pronounced sexual dimorphism in traits such as body size, immune function, and sexually selected characteristics (Sayadi et al., 2019; Chen *et al*., 2025). Several genes underlying these dimorphic traits show evidence of Y-linked translocation followed by regulatory divergence, highlighting the dynamic role of sex chromosomes in shaping sexually selected phenotypes.

Together, these genomic resources and analyses have substantially advanced our understanding of how nutrition, development, reproduction, and host adaptation evolve in Bruchinae beetles, while also revealing opportunities for sustainable pest management. As mentioned above, in *C. maculatus*, adaptation to a novel host such as lentil is driven by specific single-nucleotide variants that enhance performance but incur trade-offs, as these variants decline in frequency when populations revert to their usual host, mung bean (Messina and Gompert, 2017). Such phenotypic reversibility underscores the host-specific nature of genomic adaptation and suggests that crop rotation or host diversification could be used to reduce pest fitness. Integrative genomic approaches combining whole-genome sequencing, quantitative trait locus mapping, and transcriptomics indicate that traits such as body weight exhibit low heritability and are instead largely shaped by evolved genetic differences, including variation in cytochrome P450-mediated detoxification pathways (Rêgo et al., 2020). In *Z. subfasciatus*, rapid host shifts to chickpea induce strong transcriptional and epigenetic responses -particularly involving histone methylation- but are accompanied by reproductive costs linked to altered expression of vitellogenin and its receptor (Rodrigues et al., 2024; Cruz et al., 2025). Collectively, these findings demonstrate how genomic approaches can identify vulnerabilities in pest life-history traits, informing the development of resistant crop varieties and management strategies that constrain adaptation while reducing reliance on chemical insecticides.

## Concluding remarks

This review integrates decades of physiological, ecological, molecular, and genomic research to provide a cohesive framework for understanding how Bruchinae beetles interact with host seeds to shape nutrition, development, and adaptation. A central contribution of the literature synthesized here is the recognition that the success of Bruchinae as major seed pests depends on a finely tuned molecular and physiological toolkit. Diversified digestive enzymes, symbiotic microbes, plastic reproductive strategies, and conserved endocrine and metabolic pathways collectively enable larvae and adults to survive and reproduce within chemically defended seeds. The evolutionary arms race between legumes and Bruchinae has generated predictable counter-adaptations, such as enzyme diversification and enhanced detoxification. These traits recur across species, revealing general principles of herbivore adaptation to plant defences.

A major conceptual and methodological advance highlighted in this review is the shift from descriptive and single-gene approaches toward genome-wide analyses. The growing availability of high-quality nuclear genomes, together with population genomics, transcriptomics, and epigenomic data, has transformed our ability to identify the genetic architecture of host use, performance, and life-history trade-offs. Studies in *C. maculatus* and *Z. subfasciatus* show that adaptation to novel or unusual hosts is often reversible, constrained by genetic trade-offs, and frequently associated with fitness costs, particularly in reproduction. These findings underscore that adaptation is neither unlimited nor cost-free and provide a mechanistic basis for understanding why host shifts succeed or fail across evolutionary timescales.

Despite substantial progress in our understanding of Bruchinae biology, most research has historically emphasized ecological and evolutionary outcomes of host use, relying on life-history traits such as body size, fecundity, and generation time, while the underlying molecular mechanisms remain incompletely understood. Future advances -both in basic biology and in the development of sustainable management strategies for field and storage systems- will strongly depend on expanded access to genomic and epigenomic resources for Bruchinae species, as well as for their current and potential host plants. These knowledge gaps have direct practical consequences. From a management perspective, current control strategies still rely heavily on chemical pesticides, such as phosphine (Perkin et al., 2016), with well-documented problems of resistance evolution and environmental impact. Alternative approaches are emerging, including plant genetic manipulation to interfere with insect digestion or behaviour. Examples include the expression of *Bacillus thuringiensis* toxins or plant-derived inhibitors targeting Bruchinae digestive enzymes (Gómez-Zurita and Galián, 2005; Kumari et al., 2022), as well as the potential development of crop varieties that lack or modify VOCs required for seed detection and reproductive activation (Capizzani et al., 2024). The increasing availability of reference genomes for crops such as *P. vulgaris* makes these strategies increasingly feasible (Schmutz et al., 2014).

At the same time, recent molecular and genomic insights offer clear opportunities to move beyond reactive control toward evolution-aware pest management. Detailed knowledge of digestive enzymes, detoxification pathways, symbiotic contributions, and reproductive regulation enables the rational design of resistant cultivars, for example through stacking protease inhibitors, amylase inhibitors, lectins, or arcelins. Genomic markers associated with adaptation, insecticide resistance, and reproductive capacity can be used to monitor pest populations and anticipate their responses to novel hosts or control strategies. Moreover, insights into transcriptional plasticity and epigenetic regulation open the possibility of targeting early adaptive responses, potentially slowing or redirecting evolutionary change before it becomes genetically fixed. Together, these approaches support integrated pest management strategies that reduce reliance on chemical insecticides and promote long-term agricultural sustainability.

To fully bridge molecular mechanisms and ecological outcomes, future work will require deeper integration of genomics with nutritional biology and life-history theory. Advances in nutritional biology provide a powerful framework for understanding how Bruchinae balance the energetic costs of detoxification, evolve tolerance to antinutritional compounds, and diverge ecologically as specialists or generalists. Detoxification of seed defences such as protease inhibitors, lectins, and secondary metabolites is energetically costly, often requiring upregulation of digestive enzymes, transporters, and metabolic pathways that divert resources from growth and reproduction. Nutritional geometry (Raubenheimer and Simpson, 1997) offers a quantitative approach to examine how larvae compensate for these costs by adjusting intake, assimilation efficiency, or developmental trajectories in response to nutrient imbalances imposed by defended seeds. Within this framework, tolerance to antinutritional compounds can be viewed not only as a molecular adaptation but also as an emergent property of energy allocation strategies shaped by host quality. Specialists, which exploit a narrow range of legumes, may evolve finely tuned compensatory and detoxification responses optimized for a predictable nutritional landscape, whereas generalists likely rely on broader, more plastic metabolic responses that permit survival across hosts but at higher energetic cost (Birnbaum and Abbot, 2020). Integrating nutritional biology with genomics and life-history theory will therefore help explain how differences in detoxification capacity, compensatory feeding, and symbiont-mediated digestion translate into distinct patterns of host use, reproductive output, and ecological breadth among Bruchinae. Ultimately, this integrative perspective links molecular mechanisms to ecological outcomes, clarifying how energy compensation and tolerance evolution shape the diversification and persistence of beetle-legume interactions, while providing a robust foundation for sustainable, knowledge-driven pest control.

## Data Availability

 Genomic data for *Zabrotes subfasciatus* isolate Parental_1345 have been deposited in DDBJ/ENA/GenBank under BioProject PRJNA1328193, BioSample SAMN51307241, and WGS accession JBRALV000000000. The version described in this paper is JBRALV010000000.
